# ID 229 - Impacto das Diretrizes Atualizadas nos Resultados de Implante Transcateter de Válvula Aórtica em uma Operadora de Saúde no Brasil: estudo retrospectivo de uma década

**DOI:** 10.5327/2237-9622.2025.v34s1.229

**Published:** 2025-11-25

**Authors:** Marcus Carvalho Borin, Mateus Goncalves de Souza, Matheus del-Valle, Fernando Martin Biscione, Carina Rejane Martins, Daniel Pitchon dos Reis, Geraldo José Coelho Ribeiro, Júlia Teixeira Tupinambás, Karina de Castro Zocrato, Lélia Maria de Almeida Carvalho, Marcela Pinto de Freitas, Maria da Glória Cruvinel Horta, Mariana Michel Barbosa, Mariza Cristina Torres Talim, Sergio Adriano Loureiro Bersan, Silvana Marcia Bruschi Kelles

**Keywords:** estenose aórtica, implante transcateter de válvula aórtica, diretrizes clínicas, procedimentos cardiovasculares

## Abstract

**Introdução:**

A estenose aórtica é uma das doenças valvares mais prevalentes e graves, particularmente em populações idosas, e sua progressão pode levar à significativa morbidade e mortalidade. O implante transcateter de válvula aórtica (TAVI – do inglês, ) surgiu como uma alternativa menos invasiva à substituição cirúrgica da válvula aórtica (SAVR – do inglês, ), inicialmente indicada para pacientes com risco cirúrgico elevado. Em 2018, novas diretrizes clínicas foram introduzidas, ampliando as indicações de TAVI para pacientes com risco intermediário e até baixo. Este estudo busca avaliar o impacto dessas diretrizes atualizadas nos resultados dos procedimentos de TAVI realizados em uma operadora de saúde em Belo Horizonte, Brasil, ao longo de uma década.

**Método:**

Este é um estudo de coorte não concorrente que analisou dados secundários de pacientes diagnosticados com estenose aórtica grave e submetidos ao implante da TAVI entre 2013 e 2023. Os pacientes foram divididos em dois grupos: aqueles tratados antes de janeiro de 2019 (n=56) e aqueles tratados após essa data (n=269), permitindo a comparação dos desfechos à luz das diretrizes atualizadas pela Sociedade Brasileira de Cardiologia. Os desfechos primários incluíram mortalidade geral e principais complicações (implantação de marcapasso e acidente vascular cerebral – AVC), enquanto os desfechos secundários englobaram taxas de sucesso do procedimento e reações adversas específicas. A significância estatística foi avaliada utilizando valores de p.

**Resultados:**

Foram incluídos 325 pacientes submetidos ao implante da TAVI. Observou-se redução na taxa de mortalidade hospitalar, de 7,1% para 5,9% (p=0,735), e na mortalidade em até um ano de seguimento, de 25% para 14,9% (p=0,064), após a implementação das diretrizes de 2018. A necessidade de implantação de marcapasso diminuiu de 28,6% para 18,2% (p=0,03), enquanto a incidência de AVC reduziu de 8,9% para 5,6% (p=0,2). Alguns parâmetros mostraram benefícios estatisticamente significativos, mas todos mostram o mesmo sentido de tendência de melhora. Observou-se uma transição do uso predominante de válvulas expansíveis por balão para válvulas autoexpansíveis, associada a melhores resultados clínicos.

**Conclusão:**

As diretrizes atualizadas e os novos protocolos implementados em 2018 resultaram em melhorias significativas nos desfechos clínicos dos pacientes submetidos a TAVI. Este estudo reforça a viabilidade e a eficácia da TAVI como tratamento para estenose aórtica grave em diversos perfis de risco, destacando a importância da atualização contínua das diretrizes clínicas e do avanço tecnológico. Esses achados têm implicações relevantes para a prática clínica e para o sistema de saúde no Brasil, indicando que a adoção de novas tecnologias, associada a protocolos com critérios de elegibilidade, pode levar a melhores resultados em saúde.

